# The redox protein p66^shc^ mediates cochlear vascular dysfunction and transient noise-induced hearing loss

**DOI:** 10.1038/srep25450

**Published:** 2016-05-09

**Authors:** A. R. Fetoni, S. L. M. Eramo, F. Paciello, R. Rolesi, D. Samengo, G. Paludetti, D. Troiani, G. Pani

**Affiliations:** 1 Department of Head and Neck Surgery, Università Cattolica School of Medicine, Rome, Italy; 2 Institute of Human Physiology, Università Cattolica School of Medicine, Rome, Italy; 3 Institute of General Pathology, Università Cattolica School of Medicine, Rome, Italy

## Abstract

p66^shc^, a member of the ShcA protein family, is essential for cellular response to oxidative stress, and elicits the formation of mitochondrial Reactive Oxygen Species (ROS), thus promoting vasomotor dysfunction and inflammation. Accordingly, mice lacking the p66 isoform display increased resistance to oxidative tissue damage and to cardiovascular disorders. Oxidative stress also contributes to noise-induced hearing loss (NIHL); we found that p66^shc^ expression and serine phosphorylation were induced following noise exposure in the rat cochlea, together with markers of oxidative stress, inflammation and ischemia as indicated by the levels of the hypoxic inducible factor (HIF) and the vascular endothelial growth factor (VEGF) in the highly vascularised cochlear lateral region and spiral ganglion. Importantly, p66^shc^ knock-out (p66 KO) 126 SvEv adult mice were less vulnerable to acoustic trauma with respect to wild type controls, as shown by preserved auditory function and by remarkably lower levels of oxidative stress and ischemia markers. Of note, decline of auditory function observed in 12 month old WT controls was markedly attenuated in p66KO mice consistent with delayed inner ear senescence. Collectively, we have identified a pivotal role for p66^shc^ -induced vascular dysfunction in a common pathogenic cascade shared by noise-induced and age-related hearing loss.

Sensory neural hearing loss by exposure to noise or ototoxic drugs and age related hearing loss (ARHL) are the most common pathologies of the inner ear, which impair significantly the quality of life. Reactive Oxygen Species (ROS) are recognized as major contributors to the onset and progression of these communication disorders. In particular, the main underlying molecular mechanisms include free-radical formation and oxidative stress, however, reduced cochlear blood flow and inflammation have also emerged as key processes in recent years^1^,^2^,^3^,^4^. Oxidative stress results from an imbalance between ROS production and the cellular antioxidant defense system. This condition represents the main mechanism of cochlear damage following noise over-exposure and ARHL^3^,^5^, beside representing the leading pathway of several neurodegenerative diseases^6^. Under stress conditions, ROS levels increase and, because of their high reactivity, participate in a variety of chemical reactions leading to cell damage, necrosis, and apoptosis via oxidation of lipids, proteins, and DNA^7^. Mitochondria are generally considered to be the primary source of ROS as well as the major target for their damaging effects^8^,^9^. With this respect, several studies have revealed the role of the p66^shc^ adaptor protein in ROS production within the mitochondria and its involvement in various oxidant-related pathologies^10^,^11^,^12^. Normally p66^shc^ resides in the cytosol, while under oxidative stress (e.g., under ischemia/reperfusion insult), it is translocated trough a PKC-dependent mechanism in the mitochondria where it serves as an important source of ROS. In mitochondria, this adapter molecule has been suggested to function as a redox enzyme possibly oxidizing cytochrome *c* and generating H_2_O_2_ through its amino-terminal portion that displays sequence similarity with certain redox enzymes^13^,^14^,^15^. Accordingly, in cells lacking the p66 gene, free radicals are reduced and tolerance to oxidative stress is increased; moreover, knock-out mice lacking the p66^shc^ gene survive acute exposure to the radical generator Paraquat^10^, and, in agreement with the “free radical theory of ageing”, are resistant to several age-related diseases, including obesity^16^,^17^, atherosclerosis^18^,^19^, ischemic injury^20^,^21^, and diabetes^17^,^22^. Importantly, much of the detrimental and ageing-accelerating activity of p66^shc^ appears to involve the (micro and macro) vasculature, where p66-dependent ROS (as in the context of diabetic hyperglycemia) promote endothelial damage and dysfunction by reducing nitric oxide (NO) bioavailability, compromising vasomotor function and participating in proinflammatory cascades that lead to endothelium activation and enhanced leukocyte adhesion and atherogenesis^23^,^24^.

Cochlea hosts a complex vascular organization, comprising at least two distinct capillary networks located, respectively, within the Spiral ganglion (SGNs) and the *stria vascularis* (StV). In particular the capillaries of the StV form a blood-labyrinth barrier that regulates ion transport and fluid balance in the inner ear and is crucial for maintaining the endocochlear potential and by extension the sensory hair cell function^25^,^26^,^27^,^28^. Accordingly, vasculature-centered pathologic processes such as reduced blood flow, hypoxia and inflammation contribute to hearing impairment following acoustic trauma and most likely also to strial presbycusis^29^,^30^,^31^

The emerging role of p66^shc^ in age-related disorders associated with increased levels of oxidative stress and vascular/endothelial dysfunction, along with the strong mechanistic commonalities between hearing impairment occurring with age and auditory defects by acoustic trauma or chemical toxicity, prompted us to investigate the potential involvement of p66^shc^ in noise-induced hearing loss (NIHL) in the rat. By a combination of functional, morphological and biochemical studies, and taking advantage of the availability of a p66 deficient mouse strain, we collected here evidence for a novel p66^shc^-centered pathogenic pathway in NIHL involving cochlear oxidative stress, inflammation and impaired blood flow, the latter revealed by a robust hypoxic/angiogenic response. These findings, while provide ground for novel preventive and therapeutic approaches against common forms of hearing loss, add NIHL and ARHL to the list of p66^shc^-related disorders, likely through the same vascular mechanisms that reportedly underlie p66^shc^ roles in ischemic, atherosclerotic and diabetic vascular damage and in whole organism senescence^23^.

## Results

### Noise-induced hearing loss is accompanied by oxidative stress and by induction and phosphorylation of p66^shc^ in the rat cochlea

**Figure 1:**
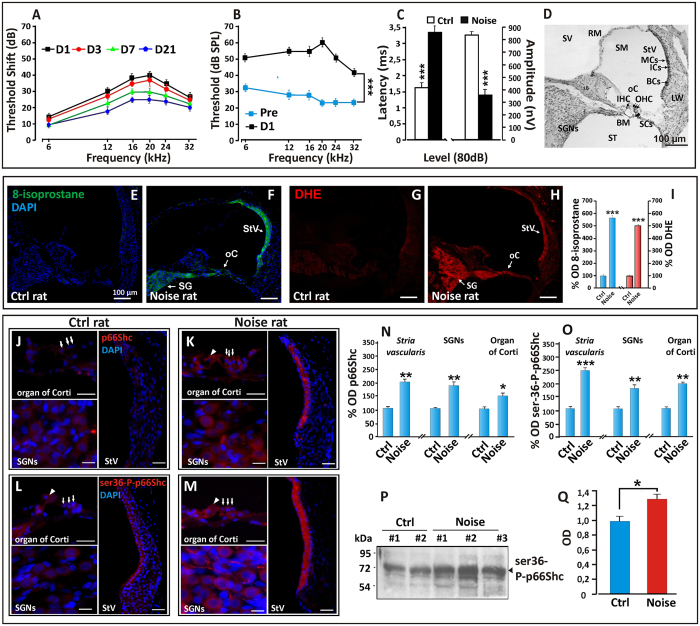
Noise-Induced hearing loss is accompanied by oxidative stress and by activation of p66^shc^ in the rat cochlea. (**A**) ABR averaged threshold shift values (±SEM) in the Noise group (n = 6 rats) showing the greatest hearing loss at day 1 (12–20 kHz) and a residual defect (~25 dB) at day 21. (**B**) Compared threshold values before and at day 1 post-noise. (**C**) Latency and amplitude of P1 wave (16 kHz, 80 dB) at day 1 post-noise. (**D**) representative H&E-stained cryosection displaying the main structures and cell types of the rat cochlea (middle turn). (**E–H**) representative confocal images of 12 μm-thick rat cochlea cryosections (12 μm), showing enhanced 8-isoprostane (**F**) and DHE (**H**) fluorescent signals in oC, SG and StV. Fluorescence accumulation indicates increased lipid peroxidation and super-oxide production at day 1 after noise exposure (compare: **E–G** with **F–H**). (**I**) quantification of fluorescence signal for 8-isoprostane (blue columns) and DHE (red columns) measured by optical density (data are normalized to control). Pictures are representative of two/three sections from each of 5 animals/group (right cochleae used for 8-isoprostane; left cochleae for DHE staining). (**J–Q**) The acoustic trauma induces p66^shc^ protein and its ser36 phosphorylated form. (**J–M**) Increased expression of p66^shc^ (compare **J** with **K**) and of its phosphorylated form ser36-P-p66^shc^ (compare **L** with **M**). Pictures are representative of 2–3 sections from each of 5 animals per group (right cochleae: p66^shc^; left cochleae: ser36-P-p66^shc^). (**N–O**) immunofluorescence analysis of total p66^shc^ and ser36-P-p66^shc^, respectively. (**P**) Anti ser36-P-p66 immunoblot analysis of total cochlear homogenates from control and noise-exposed rats. Relevant bands are indicated by arrowhead. (**Q**) ser36-P-p-66^shc^ band densitometric quantitation confirming increased phosphorylation signal in the Noise group. Values are mean ± SD of 2 controls (Ctrl) and 3 trauma-exposed (Noise) samples. Blot representative of two/three independent experiments. Statistics: (**B,C,I**) by two-way ANOVA (***p < 0.0001); (**N,O**) by one-way ANOVA and Q by two-tailed t-test (*p < 0.05; **p < 0.001; ***p < 0.0001). Scale bar: (**E–H**) 100 μm; (**J–M**) organ of Corti 30 μm, StV 50 μm, SGNs 15 μm. SV: *scala vestibuli*, RM: Reissner membrane, SM: *scala media*, MCs: marginal cells, ICs: intermediate cells, BCs: basal cells, LW: lateral wall, StV: *stria vascularis*, oC: organ of Corti, SGNs: spiral ganglion neurons, SCs: supporting cells, BM: basilar membrane, OHC: outer hair cells, IHC: inner hair cells, ST: scala *tympani*, LB: *limbus*.

In order to confirm the role of oxidative stress in NIHL and to gain initial insight in the potential role of p66^shc^ in this process we subjected Wistar rats to a paradigm of acoustic insult that models NIHL^32^,^33^. We recorded ABRs (Auditory Brainstem Responses) before and 1, 3, 7 and 21 days after noise exposure; in this experimental setting, loss of auditory function was quantified as threshold shift, i.e. the difference between the pre-noise and post-noise exposure value of auditory threshold for a given frequency. In noise-exposed rats (Fig. 1A), at day 1 after acoustic trauma the temporary threshold shift was elevated of about 30–40 dB for mid and high frequencies and of 15 dB for the low frequencies, with the greatest hearing loss occurring in the 12–24 kHz range (Fig. 1B). Three days after acoustic trauma, there was a small partial recovery in the Noise group and the average of threshold shift decreased approximately of 5 dB for all frequencies. At day 7, an additional 10 dB recovery of thresholds was detected involving all frequencies with a greater attenuation in the mid and high frequency range. At day 21 measurement, thresholds continued to recover; however, as previously reported^32^,^33^, noise-exposed rats showed a residual permanent threshold shift of about 10–25 dB for low and mid/high frequencies respectively, (Fig. 1A).

To further characterize the functional effect of exposure to noise, we studied the latency/intensity and amplitude/intensity curves of ABR waveforms at 16 kHz. The ABR latency provides insight on the transmission times along the auditory pathway, whereas ABR amplitude is a function of neural synchrony and of the number of neural units firing; their analyses can therefore provide diagnostic information on these neural functions. Evaluations of ABR P1 wave at day 1 (Fig. 1C) showed increased latency and decreased amplitude, consistent with significant impairment of the auditory function at this early time point after acoustic trauma.

Based on the above findings, we used the lipid peroxidation marker 8-isoprostane and the superoxide marker DHE (Dihydroethidium) to evaluate the levels of oxidative stress in the cochlea (HCs, SGNs and *stria vascularis,* Fig. 1D) 24 h after noise exposure (Fig. 1E–H), coincident with the maximum auditory defect. As shown in Fig. 1E,G, 8-isoprostane and DHE signals, barely detectable in the unexposed cochleae, became intense in all cochlear regions in noise-exposed animals (Fig. 1F,H and optical density in Fig. 1I), consistent with accumulation of ROS and oxidative damage in these structures.

In order to establish an initial correlation between noise-induced cochlear oxidative stress and p66^shc^ we immunostained inner ear sections isolated after 24 hours of noise exposure or *sham* treatment for p66^shc^ (Fig. 1J,K) or its ser-36 phosphorylated form (ser36-P-p66^shc^) (Fig. 1L,M). Immunofluorescence analysis displayed a faint staining for p66^shc^ (Fig. 1J and optical density in Fig. 1N) in the untreated group and a strong signal increase in the cochlea of exposed animals, in particular in the *stria vascularis* and SG (Fig. 1K). Overlapping results were obtained with ser36-P-p66, whose intensity and distribution mirrored that of total p66^shc^ (Fig. 1L,M and optical density in Fig. 1O). Accumulation of phosphorylated (i.e. active) p66^shc^ in the cochlea was also confirmed by phospho-specific western blot analysis of bulk cochlear homogenates using an anti phospho ser36-p66^shc^ specific antiserum (Fig. 1P). Band quantification, in particular, revealed for ser-36-p66 an increment of about 30% (p < 0.05 by two-tailed t-test) in Noise group compared to control rats (Fig. 1Q). Collectively, these findings indicate that p66^shc^ is expressed in the rat cochlea and is induced and phosphorylated in response to an acoustic trauma, in a fashion that parallels significant auditory loss and the establishment of intense oxidative stress.

### NIHL and oxidative stress are ameliorated in p66^shc^ deficient mice

**Figure 2:**
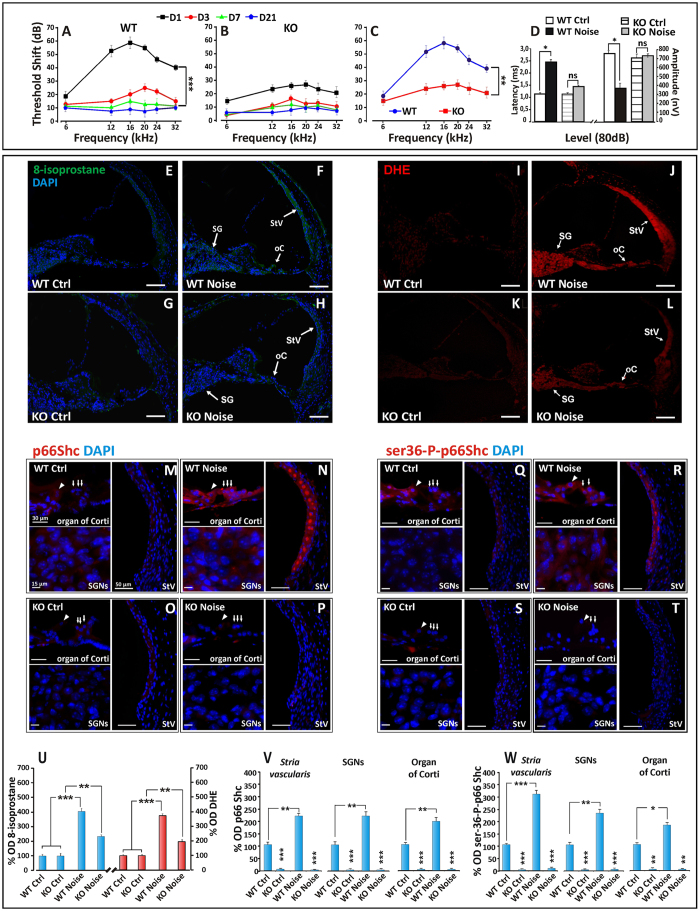
NIHL and oxidative stress are ameliorated in p66^shc^ deficient mice. (**A,B**) ABR averaged threshold shift values (±SEM,) in WT Noise (n = 6) and p66KO Noise (n = 6) groups. (**C**) Overlay of WT Noise and p66KO Noise curves at day 1, displaying significant protection (about 40 dB TS) from early NIHL in the latter mouse strain. (**D**) Latency and amplitude of P1 wave at day 1 post-noise as in Fig 1C. (**E–L**) representative confocal images of mouse (6 μm) cochlear cryosections at day 1 post-noise exposure showing enhanced 8-isoprostane (**F–H**) and DHE (**J–L**) fluorescent signal in multiple cochlear structures (arrows) including oC, SG and StV. Fluorescence accumulation in the WT mouse indicates increased lipid-peroxidation and super-oxide production (arrows) after noise exposure (compare: **E–I** with **F–J**). Trauma-induced oxidative stress is nearly abolished in the cochleae of p66KO mice (compare: **G–K** with **H–L**). Pictures are representative of two/three sections from each of 5 animals/group (right cochleae used for 8-isoprostane and left ones for DHE staining). (**M–T**) Confocal representative images of cochlear cryosections immune-stained for p66^shc^ (four panels M-P on the left) and ser36-P-p66^shc^ (four panels **Q–T** on the right); (**M,O,Q,S**) control specimens; (**N,P,R,T**) day 1 after acoustic trauma. Immunostaining for both p66^shc^ and its ser36-phosphorylated form is increased by noise in all cochlear structures of WT mice, including StV (panels **N** and **R**). No signal is detected in the corresponding cochlear structures from p66KO mice, confirming staining specificity (**P,T**). Arrows: OHC; Arrowheads: IHC. (**U**) quantification of fluorescence signal for 8-isoprostane (blue column) and DHE (red column) measured by optical density (data are normalized to control). (**V,W**) fluorescence signal quantitation for p66^shc^ and ser36-P-p66^shc^ respectively. Pictures representative of two-three sections from each of 5 animals/group (right cochleae: p66^shc^; left cochleae: ser36-P-p66^shc^). Statistics: A-D by two-way ANOVA with Tukey’s post-hoc analysis (*p < 0.05; **p < 0.001;***p < 0.0001). (**U–W**) by two-way ANOVA (*p < 0.05; **p < 0.001; ***p < 0.0001). Scale Bars: (**E–L**) 100 μm; (**M–T**) organ of Corti 30 μm, StV 50 μm, SGNs 15 μm.

To establish a mechanistic connection between p66^shc^ induction by noise and NIHL, we subjected wild type 129Sv mice and p66-deficient (p66KO) mice^10^ to acoustic trauma and evaluated ABRs at different times as described for rats. Like in rats, the greatest hearing loss occurred in the mid-high frequencies range (12–24 kHz). The baseline ABR thresholds (day 0) did not differ among the assigned treatment groups (data not shown). At day 1 after noise exposure, the threshold shift in WT mice was of about 40–60 dB for mid and high frequencies and of 20 dB for the low frequencies, with the highest threshold shift being observed at 16 kHz (p < 0.0001) (Fig. 2A). Interestingly p66KO animals recorded at the same day appeared remarkably resistant to noise, with an elevation of threshold of only about 15–25 dB across all frequencies (Fig. 2B). At 3 and 7 days after noise exposure a prompt recovery of 30–40 dB of threshold was detected in WT animals at all frequencies, with a greater attenuation in the mid and high frequency range. At day 21 measurement, thresholds continued to recover to reach a permanent shift value of about 5–10 dB for low, mid and high frequencies (Fig. 2A). Differences between WT and p66KO mice, evident at day 1 (Fig. 1C), become progressively smaller over later measurements, with threshold shift curves superimposable to WT controls at 7 and 21 days and a very similar (5–10 dB) threshold shift (Fig. 2A,B). Evaluation of ABR P1 wave at day 1 (Fig. 2D) showed increased latency and decreased amplitude in WT mice but not in KO, in keeping with the above evidence of preserved auditory function after noise exposure in the latter mutant strain.

Prompted by these findings, we compared the two strains for the levels of cochlear oxidative stress 24 h after noise exposure. As shown in Fig. 2E–L, 8-isoprostane staining and DHE signal, barely detectable in the unexposed cochleae, became intense in all cochlear structures in noise-exposed wild type animals (Fig. 2F,J). However, in keeping with functional data, fluorescence increase was markedly attenuated in p66KO exposed mice (Fig. 2H,L), suggesting that p66^shc^ is required, to a large extent, for the long reported noise-induced cochlear oxidative damage.

Consistent with these findings, and with similar evidence in rats, immunofluorescence analysis of cochlear sections from wild type mice confirmed a strong induction of both p66^shc^ (Fig. 2M,N) and ser36-P-p66^shc^ (Fig. 2Q,R), especially in the *stria vascularis* and Spiral ganglion, 24 hours after noise exposure. Importantly, this signal was specifically attributable to p66^shc^, as confirmed by lack of reactivity to either antiserum in the cochleae of Ctrl (Fig. 2O,S) and Noise p66 KO animals (Fig. 2P,T). The immunofluorescence data were confirmed by optical density analyses both for 8-isoprostane and superoxide (Fig. 2U) and for p66^shc^ and ser36-P-p66^shc^ (Fig. 2V,W) level.

### Noise induces a proinflammatory and proangiogenic response in rat cochlea

Inflammation participates in noise-induced hearing loss^1^,^2^; moreover, inflammation is a common consequence of tissue oxidative damage, and evidence exist that lack of p66^shc^ may protect tissues, at least in part, by reducing damage-associated inflammation^34^,^35^. To obtain a snapshot of the inflammatory cytokines and factors released in the cochlea in response to acute noise injury, cochlear homogenates from sham-and noise-treated rats were interrogated for the expression of multiple inflammatory mediators by means of a commercial cytokine array.

**Figure 3:**
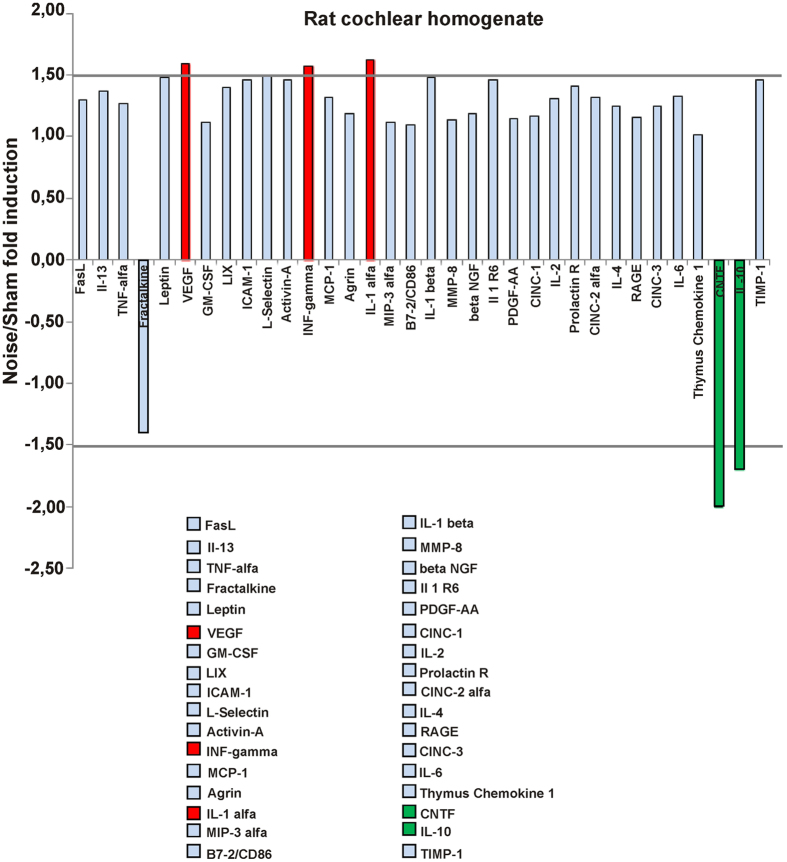
Noise induces a proinflammatory and proangiogenic response in rat cochlea. Bar histogram illustrating the differential expression of 34 cytokines in the cochlear homogenates of Noise-exposed and Sham-treated Wistar rats (pools of 5 cochleae from 5 animals/group), simultaneously evaluated by a commercial antibody array. Values are Noise-to-Sham ratios (fold induction if >1 or fold reduction, i.e. −1/fold induction, if <1) calculated from duplicate spots for each cytokine. Cytokines displaying a fold induction or a fold reduction >1.5 (with p < 0.05 by *t*-test) were highlighted in red or green, respectively. Picture representative of two independent hybridizations.

Of the 34 factors tested, only 3 (INF gamma, Interleukin 1 alpha, and VEGF) displayed a fold increase >1.5 in the noise-exposed sample; conversely, the Ciliary Neurotrophic Factor (CNTF), a trophic molecule potentially implicated in age-related hearing loss^36^, was markedly decreased (2 fold reduction) by noise (Fig. 3). Similarly decreased was the content of Interleukin 10, a cytokine broadly recognized “anti-inflammatory” or inflammation-terminating functions^37^. Overall, this analysis provided evidence for an early proinflammatory and angiogenic cochlear response to acute acoustic trauma, to be further investigated in the context of p66^shc^ deficiency.

### p66^shc^ contributes to cochlear ischemic/angiogenic response to noise

**Figure 4:**
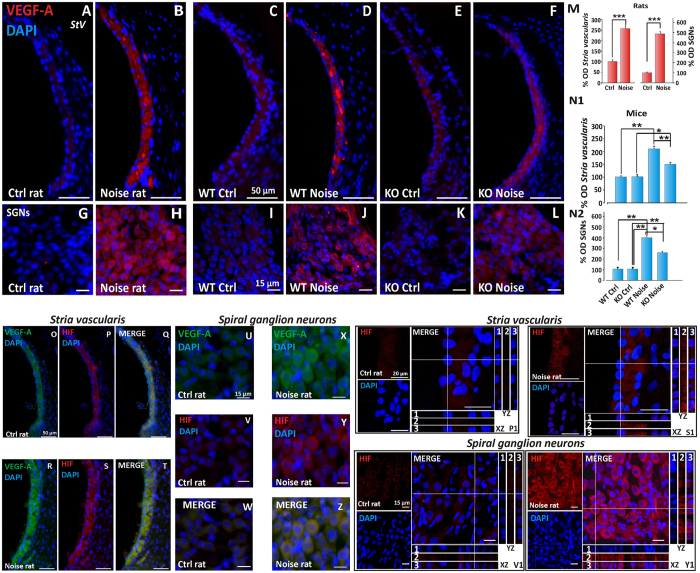
p66^shc^ contributes to a cochlear ischemic/angiogenic response to noise. (**A–L**) immunofluorescence analysis of representative cochlear sections from rats (panels **A,B,G,H**) and mice (WT and p66KO, panels **C**–**F** and **I–L**) revealing robust induction of VEGF-A in the stria vascularis and Spiral ganglion neurons (SGNs) 24 hours after noise exposure (compare **B,D** and **H,J** with **A,C** and **G,I**). VEGF increase by acoustic trauma is blunted, although not abolished, in p66KO mice compared to WT animals (compare **F,D** with **L,J**). Pictures representative of 2–3 sections from each of 5 animals per group. M, N1 and N2: Fluorescence quantitation from *n* = 9 sections/group, confirming VEGF-A increase by noise in both rats and mice and partial protection in p66KO animals compared to p66-proficient controls. Data are normalized to control. O through Z: Hypoxia Induced factor 1 alpha (HIF-1α) is induced by noise in the rat cochlea and co-localizes with VEGF in stria vascularis (**O–T**) and spiral ganglion neurons (**U–Z**). (**O,R,U,X**) VEGF-A (green fluorescence); (**P,S,V,Y**) HIF-1α (red fluorescence); (**Q,T,W,Z**) merge. HIF-1α and VEGF are both induced by noise and co-localize in all the examined structures. P1, Y1, S1, V1: VEGF activation by HIF was confirmed by confocal Z-stack analysis and HIF-1α nuclear translocation was assessed in SGNs and stria vascularis. P1, S1, V1, Y1: representative high magnification images of anti-HIF-1α/DAPI double staining and confocal Z-stack analysis XZ and YZ (box 1 = DAPI; 2 = HIF-1α; 3 = merged HIF-1α/DAPI). XZ and YZ cross-sections (referred to the dashed lines) show both cytosolic and nuclear increase of HIF-1 α fluorescence signal in noise (S1 and Y1) compared to control (P1 and V1) samples. Pictures representative of 2–3 sections from each of 5 rats per group (right cochleae for VEGF-A and left cochleae for HIF-1α). Statistics: M by one-way ANOVA (***p < 0.0001), N1 and N2 by two-way ANOVA (*p < 0.05, **p < 0.001, ***p < 0.0001). Scale Bars: in A-F StV 50 μm, in SGNs 15 μm. In O-Y1: StV O-T 50 μm, P1, S1 20 μm; SGNs U-Z 15 μm.

Prompted by the presence of VEGF-A among the cochlear cytokines induced by noise, and in order to identify the cochlear structures expressing VEGF-A, a series of immunofluorescence studies were performed in rats (Ctrl and Noise-exposed Fig. 4A,B,G,H) and in WT and p66KO mice (Ctrl and Noise-exposed, Fig. 4C–F,I–L) 24 h after the acoustic insult. In agreement with biochemical analysis of rat cochlear homogenates, noise exposure strikingly increased VEGF-A immunoreactivity in all cochlear structures, the *stria vascularis* displaying, as expected for an angiogenic factor, the most evident changes (Fig. 4B,H). Importantly, these changes were blunted in the cochleae of p66KO compared to WT mice (Fig. 4D,J,F,L). Quantitative analysis of fluorescence intensity in the *stria vascularis* and SG confirmed that VEGF-A immunostaining is significantly increased by noise both in rat and mouse cochlea, in a fashion that requires p66^shc^ and is markedly reduced in p66KO animals (Fig. 4M,N1 and N2).

Since noise insult reportedly compromises cochlear microcirculation^38^,^39^, and VEGF expression often occurs in response to tissue ischemia/hypoxia through transcriptional upregulation by the hypoxia induced factor 1 alpha (HIF-1α)^40^, we also evaluated, at the same time point, the expression of both VEGF-A and the latter hypoxic marker in cochlear sections in rats before and 24 h after acoustic trauma (Fig. 4O–T, StV; Fig. 4U–Z, SGNs). As expected, HIF-1α was nearly undetectable in the cochlea of sham-treated animals (Fig. 4P,V); remarkably, however, noise exposure led to a strong increase of HIF-1α immunoreactivity in *stria vascularis* and spiral ganglion neurons (Fig. 4S,Y), specifically in both nuclei and cytosol as shown by Z-stack confocal analyses (Fig. 4S1,Y1). Interestingly, confocal analysis of sections simultaneously stained for HIF-1α and VEGF-A (Fig. 4Q,T,W,Z) confirmed that the expression patterns of these two protein species largely overlap in noise-exposed samples (compare in Fig. 4Q,W with T,Z), suggesting that they belong to the same hypoxic signaling cascade triggered, in highly vascularized areas of the cochlea, by p66^shc^-dependent oxidative stress and microvascular dysfunction.

### Delayed ARHL in p66KO mice

**Figure 5:**
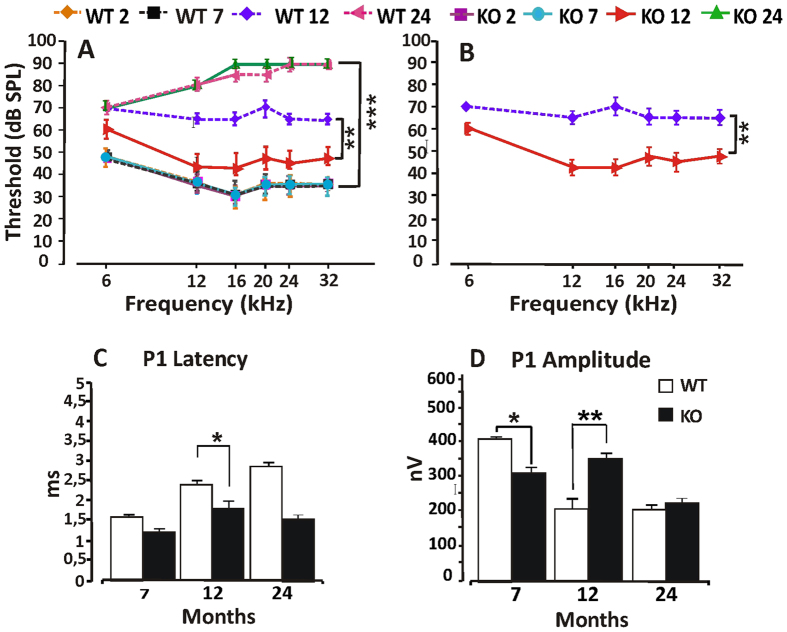
Delayed ARHL in p66KO mice. (**A,B**) ABR threshold values in dB (mean ± SEM) as recorded at 2, 7, 12 and 24 month age in WT and p66KO mice. Elevated threshold values at 12 and 24 months indicate that auditory function spontaneously declines with age in a fashion that is delayed (difference with WT at 12 months) but not prevented (no difference at 24 months) in p66KO mice across the analyzed frequencies. (**C,D**) evaluation of neural transmission through ABR latency and amplitude analysis of the waveform P1 at 16 kHz in WT and p66KO animals of 7, 12 and 24 month age. In WT mice longer latency and reduced potential amplitude over time, two functional correlates of ARHL, are already evident at 12 months, but only appear at 24 month in p66KO animals, consistent with delayed hearing loss in this strain. ABR recordings performed in 8 mice per group at the different time points. Histogram columns are Mean ± SD. Statistics by three way (**A**) and two-way ANOVA (**B–D**), followed by Tukey’s post-hoc analysis. Asterisks denote significant differences between genotype/groups (*p < 0.05, **p < 0.001, ***p < 0.0001).

Cochlear oxidative damage, inflammation and impaired blood flow are also typical features of age-related inner ear degeneration leading to presbycusis^30^,^41^,^42^. Since p66^shc^ deletion delays several features of ageing in mice, we asked whether p66KO animals could be protected by ARHL as well as by NIHL. To answer this question we performed ABR threshold measurements in WT and p66KO mice at 2, 7, 12 and 24 month old. WT and p66KO mice of age 2 and 7 month had the same threshold value across the analyzed frequencies (about 50 dB at 6 kHz and about 30–35 dB in mid-high frequencies) while, 12 month-old WT mice had significantly higher threshold values (65–70 dB across frequencies, Fig. 5A) compared to younger animals. Such time-dependent decline of auditory function was however attenuated in p66KO mice of the same age, that displayed threshold values 15–30 dB lower than WT controls, with the highest difference (42% amelioration) observed at 16 kHz (Fig. 5B). 24 month-old mice displayed a further increase in ABR thresholds (up to 80–90 dB across frequencies), with no longer difference between the WT and p66KO strains (Fig. 5A).

We further analyzed ABR waves by studying the latency/intensity and amplitude/intensity curves of ABR waveforms at 16 kHz in 7, 12 and 24 month old mice (shown in Fig. 5C,D for P1 wave). In keeping with threshold analysis, the WT latency at 12 month age was prolonged about ~1 ms for P1 as compared to p66KO mice (Fig. 5C). The WT amplitude rapidly declined with age, and, at 12 month age, was lower about ~300 nV for P1 as compared to p66KO, in which amplitude values did not significantly change compared to 7 months (Fig. 5D). However, at 24 month age there was no amplitude difference between WT and p66KO mice (Fig. 5D). These findings suggest an impairment of neural synchrony and/or a decrease in the number of neural unit firing during ageing that is similar to that observed in noise-exposed animals^43^ as shown in Figs 1 and 2. More important, they confirm that p66^shc^ deletion delays (but does not prevent) the age-dependent decline of auditory function in 129SvEv mice, possibly by impinging on the same oxidative and circulatory mechanisms that also underlie NIHL.

## Discussion

The main findings of the present study are that: a) p66^shc^ is expressed in multiple structures of rodent (mouse and rat) cochlea, and its levels of expression/phosphorylation are increased, especially in the *stria vascularis* and spiral ganglion, 24 hours after the acoustic insult; b) 129SvEv mice lacking the p66^shc^ adapter protein are resistant to the transient impairment of auditory function induced by noise in their p66^shc^-proficient controls; c) the acute noise-induced hearing loss is accompanied, in rats and mice, by histochemical and biochemical signs of oxidative stress, inflammation and compromised cochlear blood flow, which are absent or attenuated in p66-deficient mice; d) the absence of p66^shc^ delays the onset of auditory decline in 129Sv mice, a strain affected by progressive ARHL. Collectively, these evidences support a pivotal role for p66^shc^ in a pathogenic cascade shared by two common and invalidating auditory disorders, and involving, as key components, oxidative stress and vascular/endothelial dysfunction.

We have compared wild type and p66−/− (p66^shc^KO) mice, both in the 129SvEv background. The interest in this specific strain was also linked to the peculiar susceptibility of 129SvEv mice to NIHL versus ARHL. Consistent with literature^44^,^45^, we found that the 129SvEv animals were remarkably noise resistant; surprisingly, however, we observed in WT mice, but not in p66KO animals, a narrow window of vulnerability to noise exposure during the first 24 hours following acoustic insult. In wild type mice, as well as in Wistar rats, the hearing deficit was associated with evidence of oxidative stress and with increased expression of both p66^shc^ and ser-36-P-p66 in the *stria vascularis*, organ of Corti and spiral ganglion neurons. These data underlined a role of p66^shc^ in the initial acute phase of noise-induced cochlear damage even in animals showing a resistance to noise as determined, at later time points, by normal threshold values at ABR analysis and absence of oxidative stress.

The transient nature of the auditory defect observed in WT animals 24 hours after noise exposure likely reflects a short-term functional imbalance rather than a structural damage resulting for instance, from death of hairy cells^46^,^47^ or loss of fibrocytes in the lateral wall^48^,^49^. We propose that such a functional defect resides in the establishment of p66^shc^- and ROS-triggered endothelial dysfunction and in (transiently) compromised cochlear blood flow. This idea is consistent with evidence of ROS increase and of p66^shc^ accumulation and phosphorylation especially in the *stria vascularis*, a highly vascularized region of the cochlea whose capillary network underlies the blood-labyrinth barrier and participates in the ion fluxes that maintain endocochlear potential and by extension neurosensory excitability^26^,^27^. Moreover, induction in rats of inflammatory markers (like interleukin 1 alpha) known to elicit endothelial activation, and evidence in both rats and mice of elevated VEGF levels following noise exposure are consistent with endothelial stress and reduced blood flow leading to cochlear hypoxia^31^,^50^. This is further corroborated by immunofluorescence detection in the rat cochlea of accumulated Hypoxia Induced Factor (HIF-1α), a master transcriptional regulator of tissue hypoxic response which includes upregulation of VEGF^40^. A role of p66^shc^ in vascular homeostasis and in particular in (micro- and macro-) vascular disorders associated with oxidative stress has been convincingly demonstrated^51^. Importantly, these disorders are typical of ageing-associated diseases like atherosclerosis and diabetic complications^23^. In a current model, p66-triggered mitochondrial ROS reduce NO bioavailability and produce highly reactive peroxynitrite, leading to impaired vasorelaxation, accumulation of oxidative damage and vascular inflammation, that further contributes to decreased blood flow. Our findings, including blunted oxidative stress and reduced (although not abrogated) VEGF levels in noise-exposed p66KO mice, clearly suggest that p66^shc^ may be responsible of a robust albeit transient phenomenon of cochlear vascular dysfunction that dramatically impairs auditory function few hours after noise exposure in p66^shc^ proficient animals.

We have also observed that p66 deletion delays the age-related auditory decline of 129SvEv, mice, with the maximum difference between p66-decifient animals and controls being evident at twelve month age. ARHL has been previously linked to disturbances of cochlear blood flow and to inflammatory changes of the cochlea^30^,^38^,^39^. Therefore, although cochleae have not been analyzed in the context of the ARHL experiments, it is conceivable that, like in other models of age-related disease, beneficial effects of p66^shc^ ablation on ARHL reflect, at least in part, a better preserved vascular function. Thus, the same p66^shc^-centered pathogenic circuitry involving oxidative stress, vascular dysfunction with inflammatory changes and cochlear ischemia may underlie both NIHL and ARHL in 129SvEv mice. Noteworthy, literature evidence and our own data indicate that this mouse strain displays a remarkable resistance to noise-induced permanent hearing loss^44^,^45^. Thus, the transient/temporary auditory loss that we were able to detect in a very narrow temporal window of sensitivity after trauma, may represent a specific component of NIHL that shares its pathogenic mechanism (i.e. vascular dysfunction) with ARHL, to which these mice are instead prone^44^.

Finally, the bulk of data from the murine and rat model connects p66^shc^ action to biochemical evidence of inflammation. This emerging connection is particularly intriguing. In fact, a low grade chronic inflammatory reaction (parainflammation) is believed to underlie most age-related pathologic processes^52^ including ARHL^41^,^53^; our findings, while standing for a shared oxidative-inflammatory axis in NIHL and ARHL, suggest that the activation of such axis, beside or in the context of vascular dysfunction, may represent a common event downstream of p66^shc^ and by extension a general link between p66^shc^, systemic ageing and reduced health-span. This attractive hypothesis deserves further validation in additional models of age-related disease and “inflammaging”^54^.

## Materials and Methods

### Chemicals

Common laboratory chemicals were from SIGMA, unless differently stated. Antibodies used are listed below in methods where appropriate.

### Animals

Male Wistar rats with normal Preyer’s reflex and male mice SvEv129 p66^shc^A^+/+^ wild-type and p66^shc^A^−/−^ (henceforth indicated as WT and p66KO respectively) were included in this study. To investigate NIHL we used two month old Wistar rats and two month old mice randomly divided into the following: A) rat control, animals that did not received any treatment (Ctrl *n* = 20); B) rat noise, animals exposed to acoustic trauma (Noise *n* = 26); C) WT control, animals that did not received any treatment (WT Ctrl *n* = 10); D) WT noise, animals exposed to acoustic trauma (WT Noise *n* = 16); E) p66KO control, animals that did not receive any treatment (p66KOCtrl *n* = 10); F) p66 KO noise, animals exposed to acoustic trauma (p66KO Noise *n* = 16). We also analyzed the trend of hearing loss in ARHL and we used WT and p66 KO mice at age 2, 7, 12 and 24 months (8 animals x group).

All efforts were made to minimize animal suffering and to reduce the number needed for the experiments in accordance with the European Community Council Directive of November 24, 1986 (86/609/EEC). All procedures were performed in compliance with the Laboratory of Animal Care and Use Committee of the Catholic University, School of Medicine of Rome and were approved by the Italian Department of Health.

### Auditory brainstem response (ABR) recordings

ABR recordings were performed to analyze the summed responses of the synchronous firing of auditory nerve fibers and auditory brainstem neurons. Before ABR recordings, the animals were mildly anesthetized (ketamine at 35 mg/kg and medetomidine at 0.25 mg/kg for rats; ketamine at 35 mg/kg and medetomidine at 0.5 mg/kg for mice) and placed in the anechoic room. Three stainless steel recording electrodes were subcutaneously inserted posterior to the tested pinna, vertex and contralateral pinna. ABRs were collected using a computer-controlled TDT System 3 (Tucker-Davis Technologies, Alachua, FL, USA) data acquisition system with real-time digital signal processing. Tone bursts ranging from 6 to 32 kHz (rise/fall time, 2 ms; total duration, 2 ms; repetition rate, 21/s) were presented monaurally in an open field using a horn tweeter (Tucker-Davis Technologies). The responses were filtered (100–3000 Hz bandpass), digitized and averaged across 500 discrete samples at each frequency-level combination. ABRs were measured at 6–32 kHz frequencies for both species. ABRs were assessed bilaterally in all animals (mice and rats) to ensure normal hearing. ABRs were reassessed in NIHL-exposed animals at several time points (1, 3, 7 and 21 days) to evaluate the effect of noise and in aging mice (2, 7, 12 and 24 months) to monitor the age-dependent auditory decline. Thresholds were determined by decreasing tone intensity in 5 dB steps starting at 100 dB and decreasing to 0 dB or until a reliably scored ABR component was detected. Then, an ascending series of ABRs was gathered starting below this point and moving up in stimulus intensity^55^,^56^. Hearing loss was estimated by comparing ABR thresholds, recorded in the animals at day 0 (before the acoustic trauma), with ABR thresholds at the different time points (1, 3, 7 and 21 days) after the noise exposure. Thus, the ABR data are expressed in terms of threshold and threshold shift that represents the difference between the pre-noise and post-noise exposure values of each animal for NIHL experimental procedure for each group. In ARHL experiments, thresholds were compared during the aging period.

#### ABR Latencies-Amplitude (Neural Transmission Times)

ABR analysis for the study of latency and amplitude was performed in rats and mice used for NIHL study and in mice used for the ARHL experimental procedure (WT and p66KO). The rodents ABR is composed of four components (labeled from P1 to P4). These components reflect the neural activity chiefly from the auditory nerve (Fetoni *et al*.^43^). We analyzed the ABR waves at 6–32 kHz however, latency–intensity (L–I) and amplitude–intensity (A–I) curves were derived at 16 kHz frequency condition because, at this frequency, it is possible to have ABR recordings at the lowest intensity of stimulation. Thus this condition provides the clearest L–I and A–I curves. An experimenter blind to treatment conditions scored the latencies and amplitudes of the ABR waves. Another experimenter then checked the ABR scoring for reliability.

### Noise exposure

Wistar rats or WT and p66KO mice were exposed to noise in the NIHL paradigm applying a standardized acoustic trauma model, used in previous papers (35;36). Rats were deeply anesthetized, placed in a sound-proof room and exposed for 60 min to a 120 dB SPL pure tone sound at a frequency of 10 kHz. The sound was symmetrically presented in open field by a two dome tweeter (TW340 × 0, Audax) positioned 10 cm in front of the animal’s head^55^,^56^. Mice were placed in the anechoic room and exposed to a 100 dB sound pressure level SPL for 90 min at 10 kHz. The sound was presented in an open field by a dome tweeter (TW340 × 0; Audax, Chateau du Loir, France) positioned at the center of the cage. The sound was generated and amplified as previously described in Fetoni *et al*. 2014.

### Immunohistochemistry and double-labeling immunofluorescence

To assess noise-induced oxidative stress we used 8-isoprostane immunostaining and Dihydroethidium (DHE) staining. p66^shc^ involvement in NIHL was analyzed by anti p66^shc^ and anti ser36-P-p66^shc^ laboratory antibodies listed below. The animals of NIHL procedure were ABR tested before and 24 hours after noise exposure and then anesthetized and sacrificed with a lethal dose. The cochleae were quickly removed, and the samples were fixed with 4% paraformaldehyde in PBS at 4 °C and a pH 7,5. Next, the cochleae were decalcified in 10% Ethylenediaminetetraacetic acid (EDTA) incubated for 48 h in 30% sucrose, embedded in OCT, and cryosectioned at a thickness of 6 μm for mice and 12 μm for rats (Cryostat CM 1950; SLEE, Nikon, TO Italy). For superoxide detection the cochlear specimens were incubated with 1 μM DHE (Invitrogen D23107, Carlsbad, CA, USA) in PBS for 30 min at 37 °C and then coverslipped with an antifade medium (ProLong Gold, Invitrogen P36930). For the immunofluorescence analyses, the slides were incubated in a blocking solution containing 1% BSA, 0.5% Triton X-100, and 10% normal goat serum in PBS for 1 h at room temperature and then incubated overnight at 4 °C with a solution containing primary antibodies (mouse monoclonal anti-p66^shc^ [LifeSpan Bioscience, WA, USA]), anti ser36-P-p66^shc^ [Clone 6E10, Alexis, CA, USA] and anti HIF1α [Novus Bio, CO, USA], all 1:50 dilution in PBS; rabbit polyclonal anti 8-isoprostane [Oxford Biomedical Research, MI, USA] 1:100 and anti-VEGF-A ([Bioss Massachusetts, USA], 1:100 dilution in PBS). These antibodies cross reacted with mouse and rat tissues. At the end of the incubation, all slides were washed twice in PBS and incubated at room temperature for 2 h in the dark with secondary reagents (donkey anti-mouse (Alexa Fluor 546) or goat anti-rabbit (Alexa Fluor 633 or 488, IgG; Invitrogen, MI, Italy) diluted 1:400 in 0.1 M PBS. After another wash in PBS, samples were double stained with DAPI (1:500) for 15 min in the dark at room temperature. Images of anti 8-isoprostane, anti-p66^shc^, anti ser36-P-p66^shc^, anti-HIF1α (hypoxia induced factor) and anti-VEGF-A (vascular endothelial growth factor) immuno-labeled specimens were taken by fluorescence microscope (Olympus BX63, MI, Italy) and confocal microscope (TCSSP2, Leica, Germany). For HIF1α confocal Z-stacks, series of 15–20 μm-thick were acquired as images of 1024 × 1024 pixels, reordered at intervals of 0.5 μm, in order to evaluate the real extent of the nuclear and/or cytoplasmatic fluorescence in high magnifications (40×) of SGNs and StV . The optical density analysis was performed to quantify 8-isoprostane, DHE, p66^shc^, ser36-P-p66^shc^ and VEGF-A fluorescence signals (LCS Lite, Leica Confocal Software)^33^.

Control experiments were performed by omitting the primary antibody during processing of tissue randomly selected across experimental groups. Staining was absent in the hair cells (HCs) and SGNs, indicating neither spontaneous fluorescence nor non specificity of antibody. Tissues from all groups were always processed together during the procedures to limit variability related to antibody penetration, incubation time, post-sectioning age, and condition of tissue.

### Western blot analysis

Cochleae were quickly removed from the skull, the tympanic bulla was exposed, and the bony capsule of the cochlea and the lateral wall tissues were removed under stereomicroscope. The specimens from each group included the modiolus and the organ of Corti. They were collected on ice and stored at −80 °C. The cochleae were rinsed and minced in PBS, put in plastic tubes and disaggregated with a tissue homogenizer in PBS containing protease inhibitor, Homogenates were clarified by centrifugation at 18.500 g and assayed for protein content by a modified Lowry method, using a commercial kit (Priotein DC, BIORAD, MI, Italy) according to the manufacturer’s recommendations. For western blot analysis, equal amounts of protein samples were resolved by SDS–PAGE, and electrotransferred onto nitrocellulose (Protran BA 85, Whatman^®^, GE Healthcare). Equal protein loading throughout the lanes was verified by reversible Ponceau-S staining. After 2 hours blocking in TBS-T containing 5% w/v dry skim milk, membranes were incubated with the primary reagent (anti ser36-P-p66^shc^, 1:1000 dilution in TBS-T + 3% dry milk) for 16 hours at 4 °C. Immuno-complexes were visualized by a HRP-conjugates goat anti mouse antiserum followed by ECL detection. Autoradiograms were acquired using a Gel. DOC Imaging Station (BIORAD) equipped with a dedicated Software (QuantityOne), and background-subtracted band intensity quantified by the Image-J software^17^.

### Cytokine assay

Cytokine profiling of rat cochlear homogenates was performed by a commercial cytokine antibody array kit (Rat Cytokine Array C2, RayBiotech, Inc., Norcross, GA, USA), according to manufacturer’s recommendations. Briefly, 1 mg protein homogenate (in 1 ml buffer) was incubated with the array membrane at 4 °C for 16 hours; after 4 washes, the biotinylated antibody cocktail solution was added for 2 hours at RT under continuous rocking. After removal of excess antibody and four additional washes immunocomplexes were visualized by Streptavidin-HRP followed by Enhanced Chemoluminescence and autoradiography. Spots were quantified on digital images by the ImageJ software. Each Spot volume (area x mean intensity) was subtracted of the nearby background signal. For each spot of the array a ratio Noise/Sham was calculated, and ratios were further normalized for the ratio between Positive control spots to account for membrane-to-membrane signal differences. Averages of duplicate spots were calculated, and Ratios >1.5 or <0.66 (1.5 fold reduction) were considered significant.

### Statistical analysis

The results are presented as means ± standard deviation (SD) or standard error of the mean (SEM). ABR data were analyzed by means of three-way ANOVA (group/genotype x day x frequency); or two-way ANOVAs (group/genotype x frequency or time point x frequency). Latency and amplitude data were analyzed by means of two-way ANOVAs (group/genotype x decibels). When ANOVA showed significant differences, pair-wise comparisons between means were tested by Tukey’s *post hoc* testing (Statistica; Statsoft, USA). Western blot data were analyzed using unpaired two-tailed Student’s t-test. In all analyses, *p* < 0.05 was considered statistically significant.

## Additional Information

**How to cite this article**: Fetoni, A. R. *et al*. The redox protein p66^shc^ mediates cochlear vascular dysfunction and transient noise-induced hearing loss. *Sci. Rep.* **6**, 25450; doi: 10.1038/srep25450 (2016).
